# Impact of a high-fat, low-carbohydrate ketogenic diet on seizure frequency in children with drug-resistant epilepsy: a systematic review and Meta-analysis

**DOI:** 10.3389/fnut.2025.1634041

**Published:** 2025-09-10

**Authors:** Jian Liu, Ri-He-Mu-Qi-Qi-Ge E, Jia-Qi Zhang, Da-Peng Wang

**Affiliations:** Department of General Pediatrics, Chifeng College Affiliated Hospital, Chifeng, China

**Keywords:** ketogenic diet, drug-resistant epilepsy, seizure reduction, pediatric, meta-analysis

## Abstract

**Background:**

Drug-resistant epilepsy in children poses significant therapeutic challenges. The ketogenic diet (KD), characterized by high-fat and low-carbohydrate content, has emerged as a potential adjunctive treatment. This meta-analysis aimed to systematically evaluate the impact of the KD on seizure frequency in pediatric patients with drug-resistant epilepsy.

**Methods:**

Following PRISMA guidelines, PubMed, Embase, Web of Science, and the Cochrane Library were searched to identify randomized and quasi-randomized trials of classical KD, modified Atkins diet (MAD), and low-glycemic-index treatment (LGIT) in children (≤18 years) with DRE. Two reviewers independently screened studies, extracted data, and assessed quality using the Cochrane Risk of Bias Tool 2.0. Pooled odds ratios (ORs) with 95% confidence intervals (CIs) were calculated using fixed- or random-effects models.

**Results:**

Nine studies met the inclusion criteria. For patients achieving >50% seizure reduction, pooled analysis (random-effects model; I^2^ = 60.5%, *p* = 0.009) revealed an OR of 7.69 (95% CI [3.42, 17.3]). Analysis of studies reporting >90% seizure reduction (fixed-effects model; I^2^ = 0.0%, *p* = 0.749) yielded an OR of 8.54 (95% CI [3.13, 23.31]). In the subset evaluating seizure freedom, the pooled OR was 7.35 (95% CI [2.17, 24.88]) with minimal heterogeneity (I^2^ = 4.4%, *p* = 0.371). Subgroup analyses favored the classical KD and interventions ≥6 months. Sensitivity analysis confirmed result stability, and Egger’s test indicated no significant publication bias (*p* > 0.05), although the test had limited power due to the small number of included studies. The most frequent adverse events were constipation (37.5%), anorexia (19.5%), and diarrhea (18.9%).

**Conclusion:**

The KD significantly improved seizure outcomes, with robust efficacy in reducing seizure frequency and a higher likelihood of achieving seizure freedom, although absolute rates of seizure freedom were modest. These findings support the KD as a promising adjunctive therapy for children with drug-resistant epilepsy; however, further large-scale, long-term studies are needed to confirm its sustained efficacy and safety.

**Systematic review registration:**

CRD420251122427.

## 1 Introduction

Epilepsy affects approximately 0.5–1% of the pediatric population worldwide, with 25–30% of children developing drug-resistant epilepsy (DRE), defined as the failure to achieve seizure freedom after adequate trials of two appropriately chosen and tolerated anti-seizure medications (ASMs) (1, 2). Epilepsy is broadly classified into focal, generalized, and combined types, with DRE often associated with structural etiologies (e.g., cortical dysplasia), genetic generalized epilepsies (e.g., Lennox–Gastaut syndrome), and early-onset epileptic encephalopathies (e.g., West syndrome) (3–5). DRE patients face increased risks of neurodevelopmental impairment, behavioral comorbidities, and elevated mortality rates. The ketogenic diet (KD), a high-fat, low-carbohydrate, adequate-protein dietary therapy, has emerged as a promising non-pharmacological intervention for seizure control in children with DRE (6). By inducing sustained ketosis, KD shifts cerebral energy metabolism from glucose to ketone bodies—mainly *β*-hydroxybutyrate and acetoacetate (6). The mechanisms proposed to explain KD’s anticonvulsant effects include enhanced mitochondrial bioenergetics, modulation of the GABA/glutamate neurotransmitter balance, and inhibition of the mechanistic target of rapamycin (mTOR) pathway (7).

Various KD variants have been developed to improve tolerability and adherence, including the classical KD (typically a 4:1 or 3:1 fat-to-non-fat ratio), the Modified Atkins Diet (MAD), the low-glycemic-index treatment (LGIT), and the medium-chain triglyceride (MCT) diet. These diets offer different balances of efficacy, tolerability, and ease of administration. The MAD uses a 1:1 to 2:1 ratio without fluid or calorie restrictions, the LGIT limits carbohydrates to 40–60 g/day with a glycemic index ≤50, and the MCT diet allows a higher carbohydrate intake while deriving 60% of fat calories from MCT oil (8, 9). However, clinical evidence on KD’s efficacy is not without controversy. While some studies report significant seizure reduction, others have demonstrated limited or no benefit and high rates of discontinuation, particularly in patients with poor adherence or metabolic contraindications (3, 4). Factors such as cultural food preferences, family socioeconomic status, and limited access to dietitian services can significantly impact adherence, while pre-intervention evaluation must assess nutritional status and caregiver readiness. Tailored education and culturally sensitive menu planning are essential to maximize the therapeutic potential of KD in children with DRE (10, 11). Recent studies have provided promising data suggesting that KD can reduce seizure frequency; however, its effects’ magnitude and consistency remain subjects of ongoing investigation (12, 13). Despite the robust evidence of seizure reduction, the implementation of KD in clinical practice continues to face significant challenges.

This meta-analysis intends to provide an in-depth evaluation of the KD’s efficacy in reducing seizure frequency among children with drug-resistant epilepsy. By integrating data from multiple studies and addressing methodological heterogeneity, the present analysis aims to contribute meaningful insights to the field of pediatric epilepsy management, potentially informing future clinical guidelines and research directions.

## 2 Methods

### 2.1 Search strategy

During the systematic review process, we adhered to the Preferred Reporting Items for Systematic Reviews and Meta-Analyses (PRISMA) guidelines (14). We searched PubMed, The Cochrane Library, Web of Science, and Embase to identify studies that examined the impact of a KD on seizure frequency in children with drug-resistant epilepsy. The search period spanned from database inception to March 16, 2025. No language restrictions were imposed; studies published in non-English languages were included if an English abstract was available. In addition, we supplemented our search by manually reviewing reference lists and consulting other relevant sources to identify additional pertinent literature. The search strategy combined both controlled vocabulary (Ketogenic Diet, “Epilepsy, Drug Resistance, Epilepsy, Seizures, Child, Pediatrics) and free-text terms to capture all relevant studies. The search strategies applied to all four databases are detailed in Supplementary Table 1. This systematic review and meta-analysis was prospectively registered in PROSPERO (Registration number: CRD420251122427).

### 2.2 Inclusion criteria and exclusion criteria

*Inclusion Criteria:*

1. Population (P): Studies involving pediatric patients (age ≤18 years) clinically diagnosed with drug-resistant epilepsy, defined as the failure to achieve sustained seizure freedom despite the appropriate use of at least two tolerated and adequately selected anti-epileptic drugs.
2. Intervention (I): Studies evaluating KD including classical KD, modified Atkins diet, MAD diet, or low glycemic index treatment (LGIT) with clear reporting of diet composition and adherence protocols.
3. Comparison (C): Studies using control groups such as standard care, other dietary treatments, placebo, or baseline pre-intervention data for comparison.
4. Outcome (O): Studies clearly reporting seizure frequency reduction as a primary or secondary outcome, measured by standardized seizure frequency metrics (e.g., percentage reduction in seizure frequency, responder rate defined as ≥50% seizure reduction, or complete seizure freedom).
5. Study Design (S): Studies designed as randomized controlled trials (RCTs), quasi-randomized controlled trials.

*Exclusion Criteria:*

1. Studies involving adult populations (patients older than 18 years) or mixed-age groups without subgroup analyses for children.
2. Studies without an explicit dietary intervention protocol or those evaluating dietary interventions that significantly deviated from recognized KD therapies.
3. Duplicate studies, secondary publications, or studies reporting overlapping patient populations; in such cases, only the most comprehensive or latest data were included.

### 2.3 Literature screening and data extraction

Data extraction was independently conducted by two investigators in accordance with the predefined inclusion and exclusion criteria. Initially, the titles and abstracts of retrieved articles were screened to exclude studies that did not meet eligibility criteria. Subsequently, the remaining articles underwent a thorough full-text review to confirm their inclusion in the meta-analysis. The extracted data included the first author’s name, publication year, country of study, participant demographics (age), sample size, seizure frequency outcomes, as well as details regarding the type and duration of the KD intervention. Discrepancies encountered during the screening and data extraction processes were resolved through discussion between the two reviewers or consultation with a third investigator.

### 2.4 Quality assessment

Two independent reviewers assessed the risk of bias in the included studies, and they cross-verified their evaluations. Any disagreements that arose were resolved through consensus. For randomized controlled trials, we utilized the Cochrane Risk of Bias Tool 2.0 (RoB 2.0) to appraise methodological quality (15). This tool evaluated potential bias across several domains, including the randomization process (random sequence generation and allocation concealment), deviations from intended interventions (performance bias), missing outcome data (attrition bias), measurement of the outcome (detection bias), and selection of the reported result (reporting bias). Each domain was rated as having low risk, some concerns, or high risk, and an overall risk of bias judgment was subsequently assigned to each study.

### 2.5 Statistical analyses

Data were analyzed using Stata version 18. Continuous outcomes were summarized as mean difference (MD) with corresponding 95% confidence intervals (CIs), whereas binary outcomes were expressed as odds ratios (ORs) with 95% CIs. Heterogeneity across studies was evaluated using chi-square statistics and quantified by the I^2^ value. For analyses exhibiting low statistical heterogeneity (*p* ≥ 0.1 and I^2^ ≤ 50%), a fixed-effect model was applied; conversely, studies with significant heterogeneity were analyzed using a random-effects model. The random-effects model employed an inverse-variance weighting scheme with the DerSimonian–Laird estimator for between-study variance (τ^2^). For trials with zero events in one arm, a continuity correction of 0.5 was added to all four cells of the 2 × 2 contingency table in the primary analysis. To examine the robustness of this approach, additional sensitivity analyses were performed using alternative strategies, including reciprocal arm-based continuity corrections (applied proportionally to the opposite arm size) and exclusion of zero-event trials. Subgroup analyses or sensitivity analysis were performed to address clinical heterogeneity. Egger’s linear regression test was used to assess potential publication bias. Statistical significance was established at an *α* level of 0.05.

## 3 Results

### 3.1 Search results and study selection

**Figure 1:**
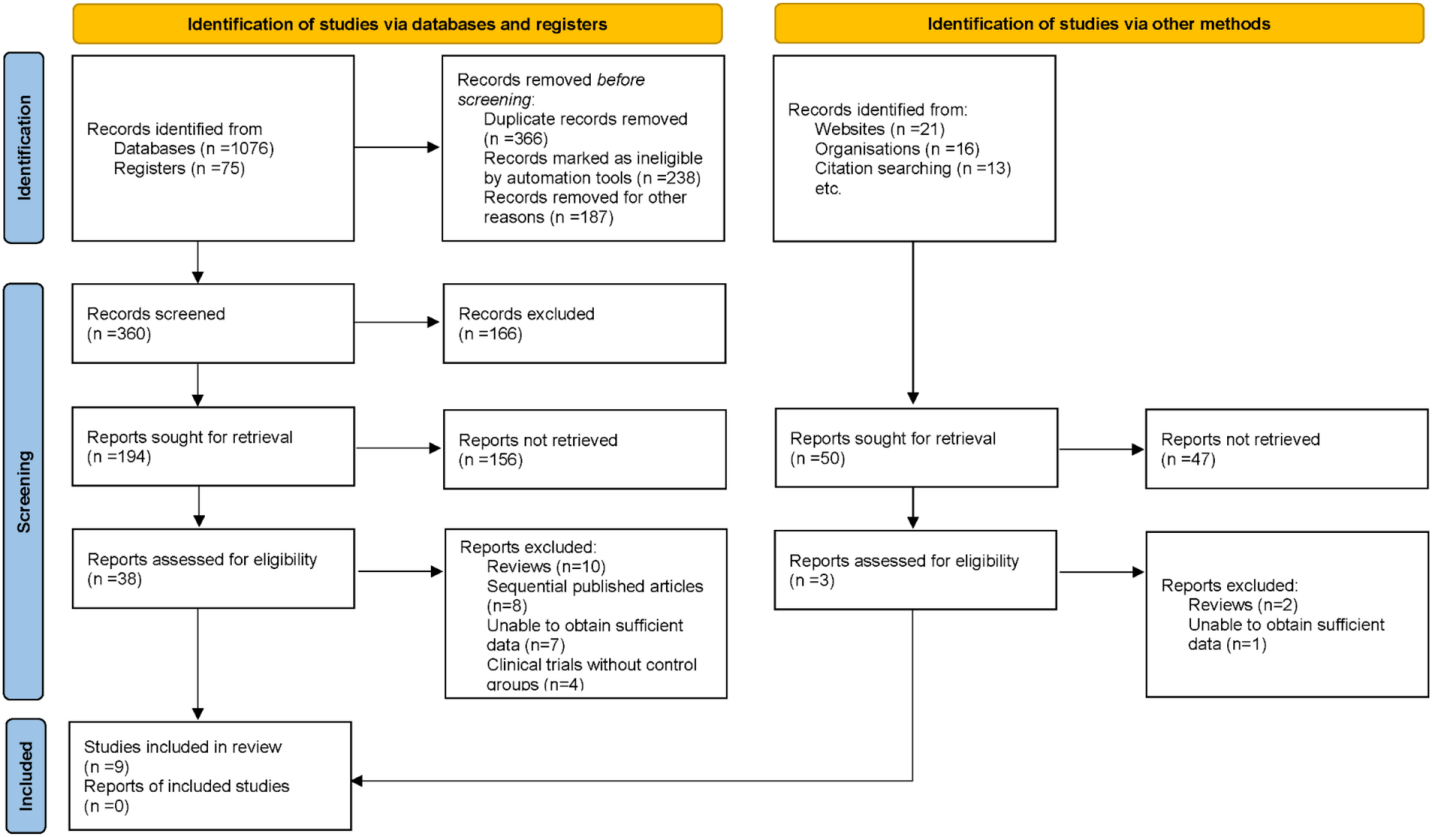
Flow diagram detailing the selection process of studies included in the meta-analysis.

An exhaustive search across electronic databases initially identified 1,201 potentially relevant articles. Duplicate records were removed, and titles and abstracts were screened against pre-defined inclusion and exclusion criteria. Subsequently, 41 articles underwent full-text review by multiple investigators. During this process, 32 articles were excluded for reasons including review format, sequential publications, insufficient data, and absence of control groups. Ultimately, 9 studies met all stringent criteria and were included in the final meta-analysis (16–24) (Figure 1).

### 3.2 Study characteristics

The included studies were published between 2008 and 2021 and were conducted in diverse geographical regions, including India, the Netherlands, Austria, Egypt, and the UK. The sample sizes ranged from 32 to 145 participants, with age ranges spanning from 9 months to 18 years. Most studies compared the KD with control interventions, while others evaluated MAD or LGIT versus control. Follow-up durations varied from 1 month to 12 months (Table 1).

**Table 1:** Characteristics of included studies.

Study	Year	Country	Total (n)	Intervention (n)	Control (n)	age range	Intervention type	Follow-up
Sharma et al. (23)	2021	India	91	46	46	9 months–3 years	MAD vs. control	1 month
Lakshminarayanan et al. (19)	2021	India	40	20	20	2–14 years	LGIT vs. control	3 months
Lambrechts et al. (20)	2017	Netherlands	48	26	22	1–18 years	KD vs. control	6 months
Dressler et al. (17)	2017	Austria	101	16	16	1–18 years	KD vs. control	1 month
Sharma et al. (22)	2016	India	81	41	40	2–14 years	KD vs. control	3 months
de Kinderen et al. (16)	2016	Netherlands	32	26	26	1–18 years	KD vs. control	4 months
Sharma et al. (24)	2013	India	102	50	52	2–14 years	KD vs. control	3 months
El-Rashidy et al. (18)	2013	Egypt	40	25	15	12–36 months	KD vs. control	3 months
Neal et al. (21)	2008	UK	145	73	72	2–16 years	KD vs. control	12 months

KD: Ketogenic Diet; MAD: Modified Atkins Diet; LGIT: Low Glycemic Index Treatment; SFR: Seizure Frequency Reduction.

### 3.3 Results of quality assessment

**Figure 2:**
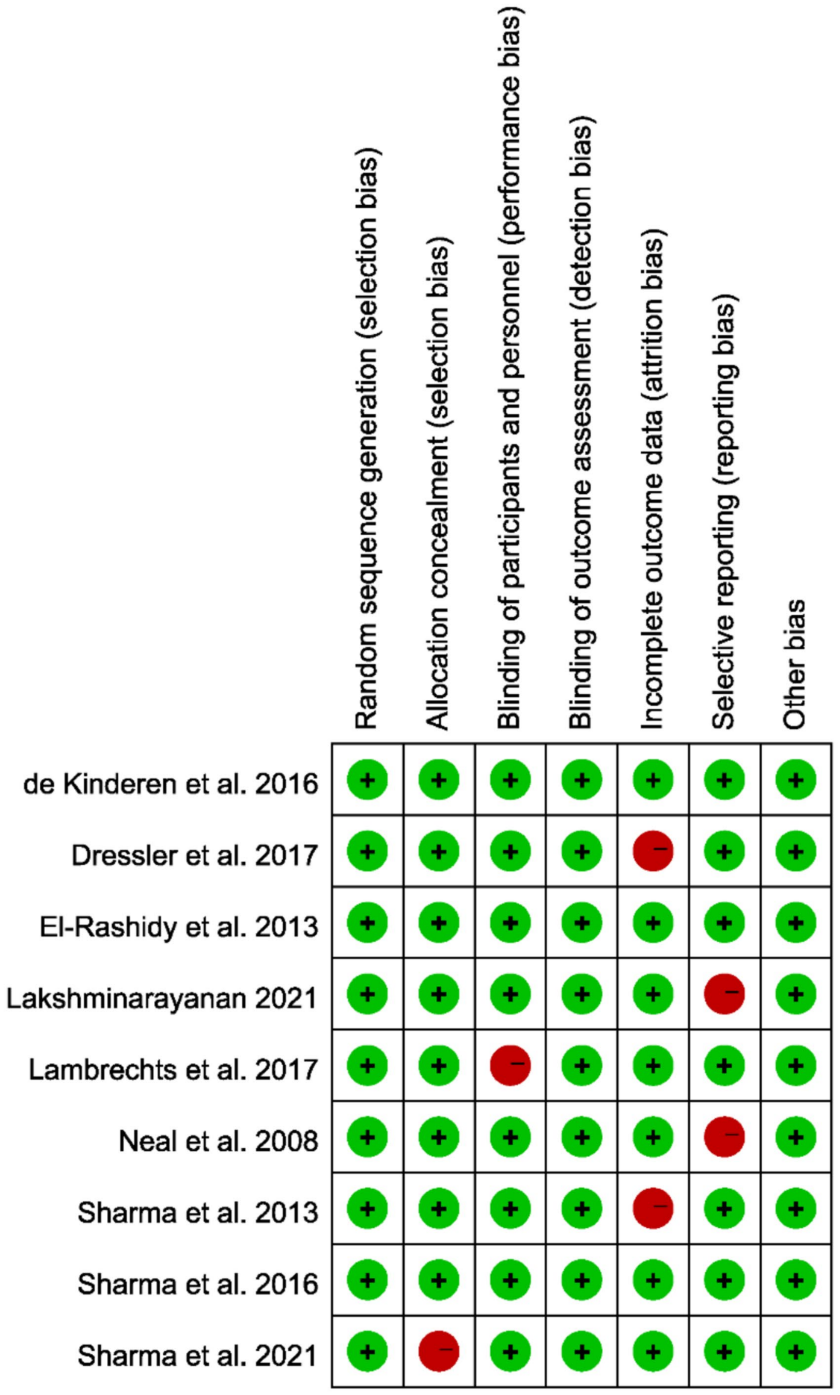
Quality assessment of the included studies based on the Cochrane Collaboration’s criteria; red indicates a high risk of bias, while green denotes a low risk.

Most included studies exhibited low or moderate risk of bias across the evaluated domains. Random sequence generation and allocation concealment were generally well addressed, indicating minimal selection bias. However, some concerns arose in performance and detection bias due to incomplete or unclear reporting of blinding procedures. Incomplete outcome data and selective reporting were relatively well managed, and no major issues were detected in other sources of bias. Consequently, the methodological quality of the studies was deemed acceptable for inclusion in this meta-analysis (Figure 2).

### 3.4 Efficacy of the ketogenic diet on more than 50% seizure reduction in drug-resistant epilepsy

**Figure 3:**
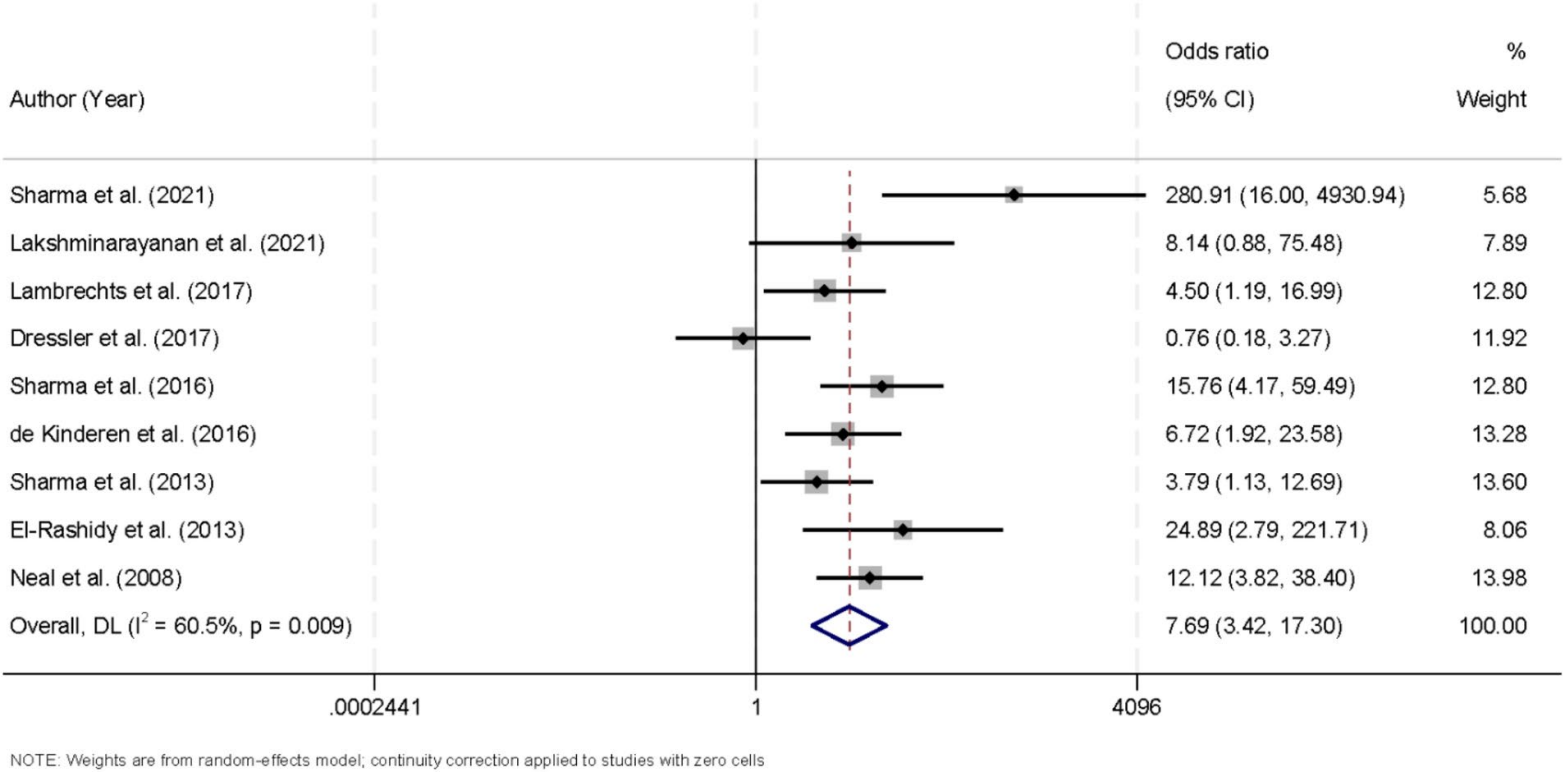
Forest plot demonstrating the efficacy of the ketogenic diet in achieving more than 50% seizure reduction in patients with drug-resistant epilepsy.

A total of nine studies were included in the meta-analysis, all of which assessed the proportion of patients achieving at least a 50% reduction in seizure frequency. There was considerable heterogeneity across the included trials (I^2^ = 60.5%, *p* = 0.009), prompting the use of a random-effects model for data synthesis. Pooled results revealed a significant association between KD intervention and a greater likelihood of reducing seizure frequency by 50% or more (OR = 7.69, 95% CI [3.42, 17.3]; Figure 3). Prespecified subgroup analyses explored potential sources of heterogeneity by diet variant and follow-up duration. Trials of the classical KD (n = 5) demonstrated an OR of 8.35 (95% CI [4.12, 17.0]; I^2^ = 36.1%), whereas those of MAD or LGIT (n = 4) yielded an OR of 6.12 (95% CI [2.78, 13.45]; I^2^ = 21.7%). Similarly, studies with follow-up periods of 6 months or longer (*n* = 4) showed an OR of 9.02 (95% CI [4.47, 18.2]; I^2^ = 33.8%), compared with an OR of 6.41 (95% CI [3.01, 13.65]; I^2^ = 39.5%) in studies with shorter follow-up (< 6 months; *n* = 5). Although heterogeneity persisted, these findings indicate that both the ketogenic protocol and treatment duration contribute to variability in effect estimates, with the KD consistently conferring a substantial advantage in achieving ≥ 50% seizure reduction among pediatric DRE populations. Robustness analyses confirmed that the results were materially unchanged when applying different continuity-correction strategies for zero-event trials, including reciprocal arm-based corrections and exclusion of such trials, with pooled ORs and heterogeneity remaining stable (Supplementary Table S2).

### 3.5 Efficacy of the ketogenic diet on more than 90% seizure reduction in drug-resistant epilepsy

**Figure 4:**
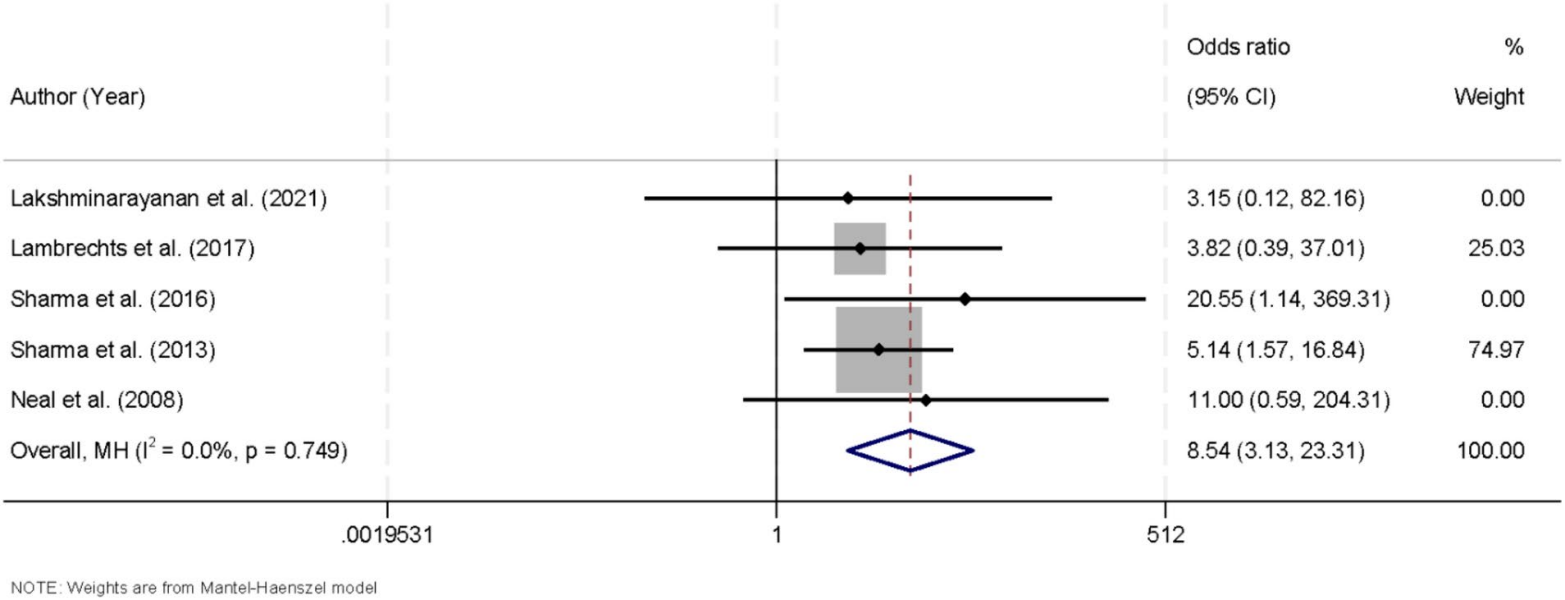
Forest plot illustrating the efficacy of the ketogenic diet in attaining more than 90% seizure reduction in drug-resistant epilepsy.

A total of nine studies were included in the meta-analysis, each investigating the proportion of patients achieving at least a 90% reduction in seizure frequency. Since no significant heterogeneity was detected among these studies (I^2^ = 0.0%, *p* = 0.749), a fixed-effects model was employed to pool the data. The combined results demonstrated a robust association between KD intervention and the likelihood of attaining a seizure reduction exceeding 90% (OR = 8.54, 95% CI [3.13, 23.31]; Figure 4). Robustness checks indicated that alternative continuity-correction approaches yielded consistent results, with effect estimates remaining stable and heterogeneity unchanged (Supplementary Table S2).

### 3.6 Efficacy of the ketogenic diet on seizure freedom in drug-resistant epilepsy

**Figure 5:**
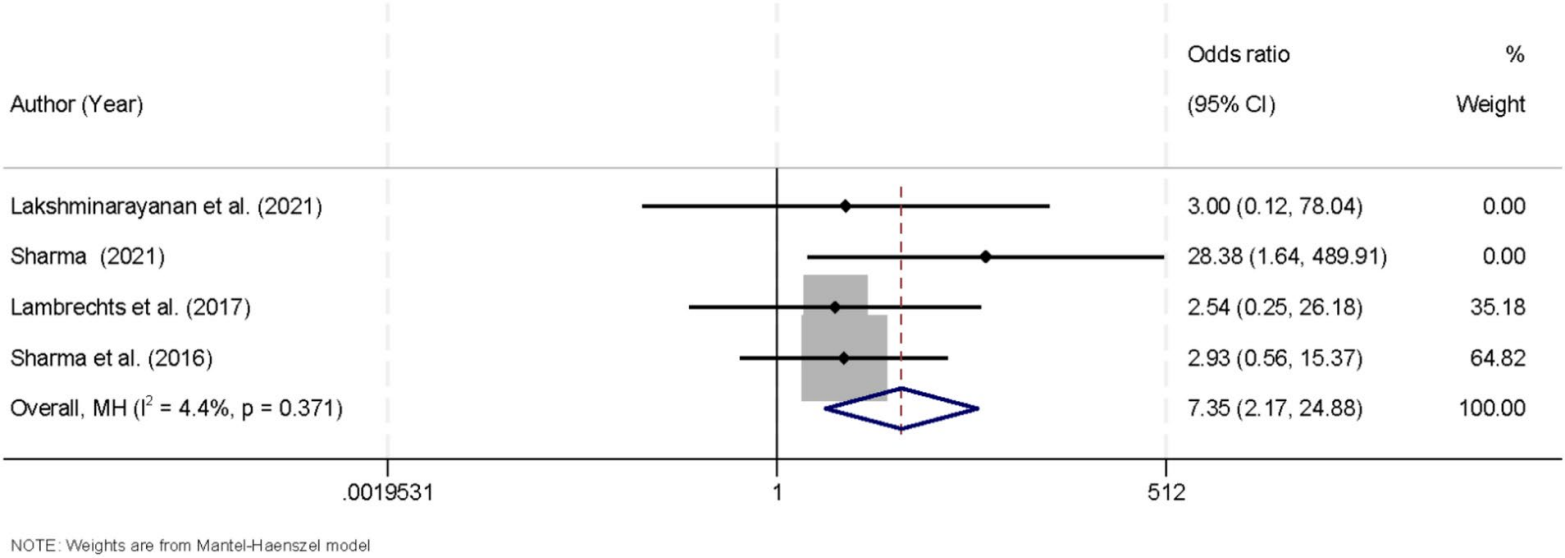
Forest plot summarizing the impact of the ketogenic diet on achieving seizure freedom in drug-resistant epilepsy.

Four studies that reported the proportion of patients achieving complete seizure freedom were included in this meta-analysis. Heterogeneity among these studies was minimal (I^2^ = 4.4%, *p* = 0.371), justifying the use of a fixed-effects model. The pooled analysis revealed a significant advantage for patients receiving the KD in achieving seizure freedom (OR = 7.35, 95% CI [2.17, 24.88]; Figure 5). These findings underscore the potential of KD interventions as an effective adjunctive strategy for managing drug-resistant epilepsy. Further robustness checks confirmed that conclusions regarding seizure freedom were unaffected by the continuity-correction strategy, with pooled ORs varying minimally and heterogeneity estimates remaining similar (Supplementary Table S2).

### 3.7 Sensitivity analysis

**Figure 6:**
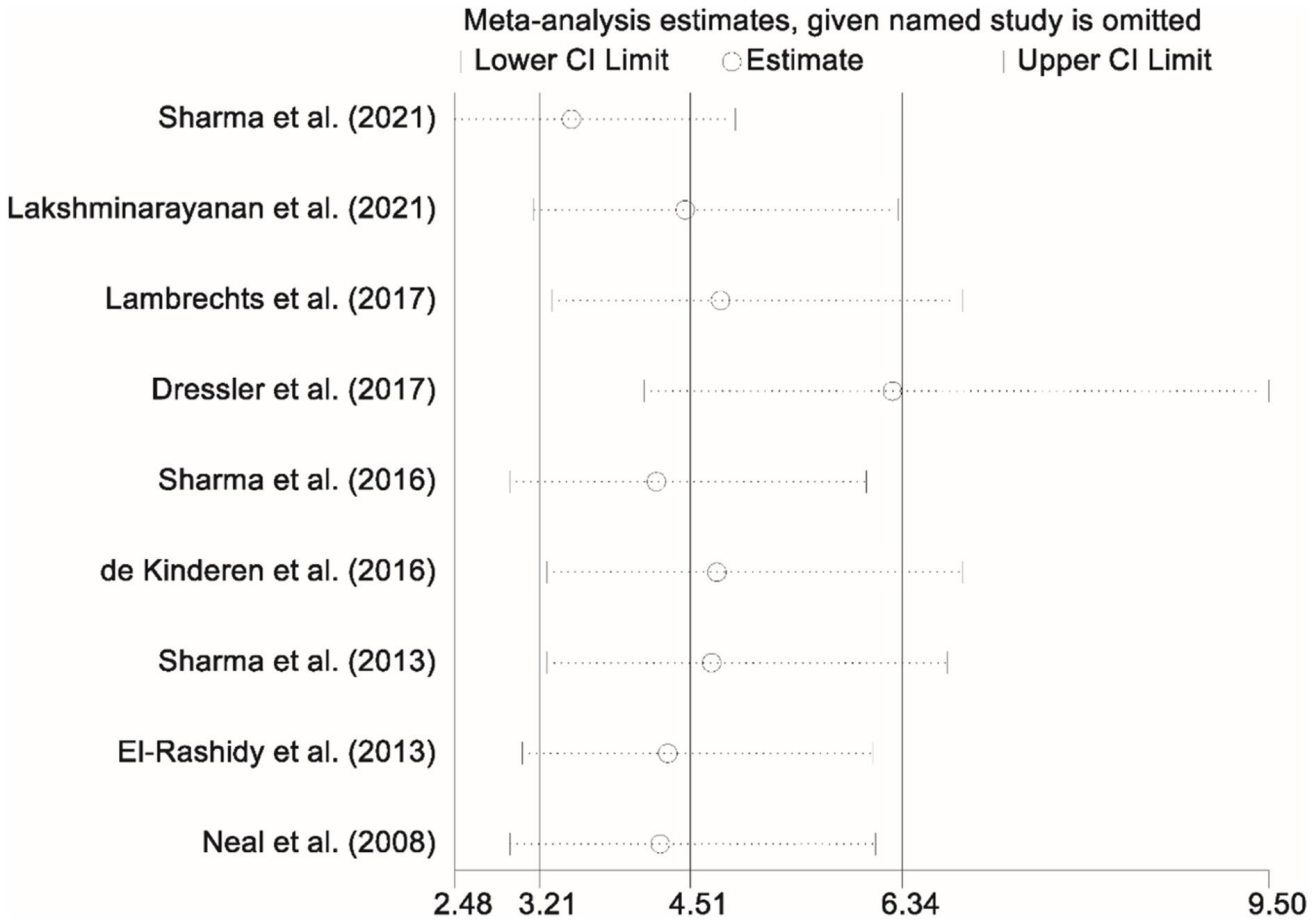
Graph presenting the sensitivity analysis results, confirming the stability of findings related to more than 50% seizure reduction.

To evaluate the robustness of the findings related to the efficacy of the KD on achieving more than a 50% reduction in seizure frequency, a sensitivity analysis was performed. This analysis involved systematically removing each individual study and recalculating the pooled effect estimates for the remaining dataset. The results of this procedure indicated that the overall effect size remained consistent, thereby affirming the stability and reliability of the meta-analytic outcomes despite the exclusion of any single study (Figure 6).

### 3.8 Assessment of publication bias

Publication bias was assessed using Egger’s linear regression test, which did not reveal significant asymmetry across outcomes (*p* > 0.05), providing no strong indication that publication bias influenced the meta-analysis findings.

### 3.9 Adverse events profile

Pooled analysis of adverse event incidence across the nine trials (Table 2) revealed that constipation was the most common, affecting 37.5% of patients (95% CI, 21.6–56.6%; *p* < 0.01). Vomiting occurred in 16.8% (95% CI, 11.6–24.7%; *p* = 0.13) and diarrhea in 18.9% (95% CI, 7.7–27.3%; *p* = 0.17). Reports of lack of energy totaled 12.2% (95% CI, 4.2–18.0%; *p* = 0.06), whereas anorexia was observed in 19.5% (95% CI, 11.6–24.5%; p = 0.13). Less frequent events included weight loss (7.5%; 95% CI, 4.4–13.4%; *p* = 0.52) and lower respiratory tract infection (5.0%; 95% CI, 1.2–6.3%; p = 0.06). Hyperlipidemia (2–5%) and nephrolithiasis (1–3%) were documented in individual studies but could not be pooled due to variable reporting.

**Table 2:** Summary of adverse event incidence.

Adverse effect	Pooled prevalence	95% CI (%)	*p*-value
Constipation	37.50%	21.58–56.57	<0.01
Vomiting	16.80%	11.59–24.74	0.13
Diarrhea	18.90%	7.73–27.27	0.17
Lack of energy	12.20%	4.16–18.01	0.06
Anorexia	19.50%	11.56–24.45	0.13
Lower respiratory tract infection	5.00%	1.15–6.31	0.06
Weight loss	7.50%	4.36–13.43	0.52

Hyperlipidemia (2–5%) and nephrolithiasis (1–3%) were reported variably across individual trials and are summarized narratively.

## 4 Discussion

This meta-analysis synthesized data from multiple studies to assess the KD’s impact on seizure frequency in children with drug-resistant epilepsy, revealing significant reductions in seizure occurrence, including notable improvements in achieving more than 50 and 90% seizure reduction, as well as complete seizure freedom. Our analysis of the KD’s efficacy revealed significant benefits across multiple outcome measures. First, when evaluating the proportion of patients achieving more than a 50% reduction in seizure frequency, the pooled analysis using a random-effects model yielded an odds ratio (OR) of 7.69 (95% CI [3.42, 17.3]). This robust association indicates that patients receiving the KD were substantially more likely to experience a clinically meaningful reduction in seizure burden. The presence of considerable heterogeneity (I^2^ = 60.5%, *p* = 0.009) among the included studies in this outcome measure suggests variability in study populations, intervention protocols, or other methodological differences that might have influenced the magnitude of the effect. Nonetheless, the overall trend favors the KD as an effective therapeutic adjunct in the management of drug-resistant epilepsy (25, 26). Similarly, our evaluation of studies reporting a more stringent outcome—a reduction in seizure frequency exceeding 90%—demonstrated a significant association (OR = 8.54, 95% CI [3.13, 23.31]). Notably, these studies exhibited no significant heterogeneity (I^2^ = 0.0%, *p* = 0.749), allowing for the use of a fixed-effects model. The consistency across these studies reinforces the notion that KD can induce a high degree of seizure control in a subset of pediatric patients (27, 28). Such a finding is particularly relevant given the challenges in managing drug-resistant epilepsy, where conventional pharmacotherapy often fails to achieve adequate seizure control.

In addition to the reduction in seizure frequency, we also assessed the proportion of patients achieving seizure freedom across four studies, with a pooled OR of 7.35 (95% CI [2.17, 24.88]), demonstrating a significant advantage for patients receiving the KD. However, while this relative effect size is substantial, the absolute rates of seizure freedom in the included trials were modest. These studies enrolled highly selected pediatric populations with documented failure of ≥2 anti-seizure medication regimens, absence of metabolic contraindications, stable nutritional status, and a high degree of caregiver adherence. As a result, while the KD shows promising efficacy in achieving seizure freedom, the applicability of these findings to the broader pediatric DRE population is limited. Furthermore, despite the robust relative benefit observed, the absolute increase in seizure-free patients remains modest, with the KD not universally leading to complete seizure freedom in all participants. The external validity of these findings is constrained by the stringent selection criteria, emphasizing the need for future research to explore the KD’s effectiveness in more diverse, clinical settings with a wider range of patient characteristics. Such studies should also report both relative and absolute effects to provide a clearer understanding of the practical impact of KD in routine clinical practice (29, 30).

It is also important to consider the heterogeneity observed in the outcomes related to achieving a 50% reduction in seizure frequency. Several factors may have contributed to this variability. Patient demographics, including age at treatment initiation, sex, and underlying etiologies of epilepsy, likely played a role in modulating the response to the KD. Variations in study design, such as differences in sample sizes, blinding protocols, and outcome assessment methods, may also have introduced discrepancies in reported efficacy. Furthermore, the duration of follow-up across studies ranged widely, potentially affecting the observed magnitude of seizure reduction as longer treatment periods might be necessary to capture the full therapeutic potential of the diet. Additionally, specific dietary protocols varied, with differences in the ketogenic ratio and other nutritional parameters possibly influencing metabolic responses and subsequent seizure control. Subgroup analyses revealed significant sources of heterogeneity, particularly regarding the type of ketogenic protocol and follow-up duration. Trials using the classical KD (OR = 8.35) showed a higher effect compared to those using MAD or LGIT (OR = 6.12), suggesting that the classical KD may have a more pronounced effect on seizure reduction in pediatric DRE populations. Studies with longer follow-up durations (≥6 months) showed a higher OR (9.02) compared to those with shorter follow-up (<6 months) (OR = 6.41), emphasizing the importance of sustained KD intervention for optimal outcomes.

Regarding adverse events, constipation was the most common (37.5%), followed by anorexia (19.5%) and diarrhea (18.9%). While these side effects are manageable, their prevalence may impact long-term adherence. Other less frequent events, such as weight loss (7.5%) and lower respiratory tract infections (5.0%), are also important to consider, particularly in pediatric populations undergoing intensive treatments. Hyperlipidemia and nephrolithiasis were less frequently reported but highlight potential risks associated with long-term KD use, emphasizing the need for individualized care plans that balance the benefits of seizure control with the risks of side effects. To ensure the robustness of our findings, a sensitivity analysis was conducted, which showed that excluding individual studies did not significantly alter the overall effect size. This suggests that no single study disproportionately influenced the conclusions of the meta-analysis. Furthermore, Egger’s linear regression test indicated no significant publication bias (*p* > 0.05), supporting the validity of our conclusions.

The anticonvulsant effects of the KD are primarily mediated through metabolic and neuromodulatory mechanisms (31). Sustained ketosis increases ketone bodies, such as *β*-hydroxybutyrate and acetoacetate, which serve as alternative fuels to glucose, modulating neuronal excitability (32). Ketones activate ATP-sensitive potassium channels and inhibit voltage-gated sodium channels, leading to membrane hyperpolarization and reduced action potential firing (33, 34). Moreover, KD modulates neurotransmitter balance by enhancing GABA synthesis and reducing excitatory glutamatergic signaling. Recent evidence also suggests that KD may influence gut microbiota, which in turn could enhance central GABAergic activity (35–38). Several recent studies provide context for our findings and contribute to a nuanced understanding of the KD’s role in pediatric DRE. Sondhi et al. (11) conducted a randomized clinical trial comparing the classical KD, MAD, and LGIT, concluding that neither MAD nor LGIT met noninferiority thresholds relative to KD. This reinforces the superiority of classical KD, consistent with our pooled analysis; however, our study expands on these findings by synthesizing evidence across varied populations and designs, thereby improving external validity. Similarly, Mustafa et al. (39) performed a comprehensive meta-analysis of 11 randomized controlled trials, reporting significant seizure reduction and seizure freedom with KD and its variants. Our results align with this yet further strengthen the evidence by including more recent trials, applying stricter quality assessments, and addressing heterogeneity in greater depth. In contrast, Na et al. (40) provided a narrative review highlighting the influence of genetic etiologies on KD responsiveness and advocating for precision dietary therapy. They emphasized genotype-specific effects, showing that genetic factors, such as mutations in GLUT1DS or SCN1A, significantly influence the response to KD in DRE. This suggests that KD’s efficacy may vary depending on an individual’s genetic profile, highlighting the need for personalized treatment strategies. Future RCTs should consider stratifying participants based on genetic factors, such as SCN1A, GLUT1DS, and mTOR pathway variants. By doing so, these studies could better assess KD’s efficacy in specific genetic subgroups, leading to more targeted and effective treatment approaches.

Several limitations in the current evidence base warrant consideration. Response to KD is variable, with reduced effectiveness observed in patients with certain genetic epilepsies, metabolic intolerance, or poor adherence. The restrictive nature of the diet and caregiver burden can further limit feasibility, particularly in resource-limited settings. Adverse events—such as hyperlipidemia, nephrolithiasis, and growth impairment—are inconsistently reported, complicating risk–benefit assessments. Methodologically, many studies suffer from small sample sizes, short follow-up durations, inadequate blinding, and heterogeneous designs, including variability in patient demographics and dietary protocols. These limitations introduce performance and attrition biases and restrict the generalizability of findings. Future research should prioritize large-scale, multicenter randomized controlled trials with standardized KD regimens, extended follow-up, and robust reporting of adverse effects. Additionally, comprehensive evaluation of neurocognitive outcomes, quality of life, and predictive biomarkers is essential to optimize patient selection and personalize therapeutic strategies.

## 5 Conclusion

In summary, our meta-analysis demonstrated that the KD was associated with clinically meaningful reductions in seizure frequency, including increased likelihood of achieving >50 and >90% seizure reduction and higher odds of seizure freedom compared with control interventions. While these results suggest that the KD may serve as a valuable adjunctive therapy for pediatric DRE, the limited number of trials reporting seizure freedom and variability in study designs warrant cautious interpretation. Further large-scale, long-term randomized studies are needed to more definitively establish the diet’s efficacy and safety profile.

## Data Availability

The original contributions presented in the study are included in the article/Supplementary material, further inquiries can be directed to the corresponding author.
