# Supplementary material for: Impact of a high-fat, low-carbohydrate ketogenic diet on seizure frequency in children with drug-resistant epilepsy: a systematic review and Meta-analysis
Source: Front Nutr. 2025 Sep 10;12:1634041. doi: 10.3389/fnut.2025.1634041 (PMC12459275; doi:10.3389/fnut.2025.1634041)
Supplement: Supplementary file 1 [file Table_1.DOCX]

Supplementary Table 1. Search Strategy for Four Databases.

| Database | Search Strategy |
| --- | --- |
| PubMed | ("Ketogenic Diet"[Mesh] AND "Epilepsy"[Mesh] AND "Drug Resistance, Epilepsy"[Mesh] AND "Seizures"[Mesh] AND ("Child"[Mesh] OR "Pediatrics"[Mesh])) OR ("ketogenic diet" AND "drug-resistant epilepsy" AND seizures AND (child OR pediatric)) |
| Embase | ('ketogenic diet'/exp AND 'epilepsy'/exp AND 'drug resistant epilepsy'/exp AND 'seizure'/exp AND ('child'/exp OR 'pediatrics'/exp)) OR ('ketogenic diet' AND 'drug-resistant epilepsy' AND seizures AND (child OR pediatric)) |
| Web of Science | TS=(“ketogenic diet” AND (“epilepsy” OR “drug-resistant epilepsy”) AND “seizures” AND (child OR pediatric)) |
| Cochrane Library | ("Ketogenic Diet":MeSH OR "ketogenic diet") AND ("Epilepsy":MeSH OR "epilepsy") AND ("Drug Resistance, Epilepsy":MeSH OR "drug-resistant epilepsy") AND ("Seizures":MeSH OR "seizures") AND (("Child":MeSH OR "Pediatrics":MeSH) OR (child OR pediatric)) |
