# Supplementary material for: Impact of a high-fat, low-carbohydrate ketogenic diet on seizure frequency in children with drug-resistant epilepsy: a systematic review and Meta-analysis
Source: Front Nutr. 2025 Sep 10;12:1634041. doi: 10.3389/fnut.2025.1634041 (PMC12459275; doi:10.3389/fnut.2025.1634041)
Supplement: Supplementary file 2 [file Table_2.DOCX]

Supplementary Table S2. Sensitivity analyses of pooled effects under different continuity-correction strategies for zero-event trials.

| Outcome | Continuity-Correction Strategy | Pooled OR (95% CI) | I² (%) | Notes |
| --- | --- | --- | --- | --- |
| ≥50% seizure reduction (9 studies) | Standard 0.5 correction (all cells) | 7.69 (3.42–17.3) | 60.5 | Primary analysis |
|  | Reciprocal arm-based correction (treatment-arm continuity method) | 7.42 (3.18–16.9) | 59.2 | Robustness check |
|  | Excluding zero-event trials | 7.88 (3.50–17.8) | 61.0 | Robustness check |
| ≥90% seizure reduction (9 studies) | Standard 0.5 correction | 8.54 (3.13–23.3) | 0.0 | Primary analysis |
|  | Reciprocal arm-based correction | 8.33 (3.01–22.8) | 0.0 | Robustness check |
|  | Excluding zero-event trials | 8.60 (3.20–23.6) | 0.0 | Robustness check |
| Seizure freedom (4 studies) | Standard 0.5 correction | 7.35 (2.17–24.9) | 4.4 | Primary analysis |
|  | Reciprocal arm-based correction | 7.12 (2.08–24.2) | 4.8 | Robustness check |
|  | Excluding zero-event trials | 7.41 (2.15–25.1) | 5.1 | Robustness check |

Standard 0.5 correction: continuity correction of 0.5 applied to all four cells of the 2×2 table.

Reciprocal arm-based correction: continuity correction proportional to the reciprocal of the opposite treatment arm size.

Excluding zero-event trials: sensitivity analysis excluding studies with zero events in both arms.
